# Exhaled breath compositions under varying respiratory rhythms reflects ventilatory variations: translating breathomics towards respiratory medicine

**DOI:** 10.1038/s41598-020-70993-0

**Published:** 2020-08-24

**Authors:** Pritam Sukul, Jochen K. Schubert, Karim Zanaty, Phillip Trefz, Anupam Sinha, Svend Kamysek, Wolfram Miekisch

**Affiliations:** 1 Rostock Medical Breath Research Analytics and Technologies (ROMBAT), Department of Anaesthesiology and Intensive Care, University Medicine Rostock, Schillingallee 35, 18057 Rostock, Germany; 2 Institute for Clinical Chemistry and Laboratory Medicine, University Clinic Carl Gustav Carus, Fetscherstr. 74, 01307 Dresden, Germany

**Keywords:** Physiology, Biomarkers, Medical research

## Abstract

Control of breathing is automatic and its regulation is keen to autonomic functions. Therefore, involuntary and voluntary nervous regulation of breathing affects ventilatory variations, which has profound potential to address expanding challenges in contemporary pulmonology. Nonetheless, the fundamental attributes of the aforementioned phenomena are rarely understood and/or investigated. Implementation of unconventional approach like breathomics may leads to a better comprehension of those complexities in respiratory medicine. We applied breath-resolved spirometry and capnometry, non-invasive hemodynamic monitoring along with continuous trace analysis of exhaled VOCs (volatile organic compounds) by means of real-time mass-spectrometry in 25 young and healthy adult humans to investigate any possible mirroring of instant ventilatory variations by exhaled breath composition, under varying respiratory rhythms. Hemodynamics remained unaffected. Immediate changes in measured breath compositions and corresponding variations occurred when respiratory rhythms were switched between spontaneous (involuntary/unsynchronised) and/or paced (voluntary/synchronised) breathing. Such changes in most abundant, endogenous and bloodborne VOCs were closely related to the minute ventilation and end-tidal CO_2_ exhalation. Unprecedentedly, while preceded by a paced rhythm, spontaneous rhythms in both independent setups became reproducible with significantly (*P-*value ≤ 0.005) low intra- and inter-individual variation in measured parameters. We modelled breath-resolved ventilatory variations via alveolar isoprene exhalation, which were independently validated with unequivocal precision. Reproducibility i.e. attained via our method would be reliable for human breath sampling, concerning biomarker research. Thus, we may realize the actual metabolic and pathophysiological expressions beyond the everlasting in vivo physiological noise. Consequently, less pronounced changes are often misinterpreted as disease biomarker in cross-sectional studies. We have also provided novel information beyond conventional spirometry and capnometry. Upon clinical translations, our findings will have immense impact on pulmonology and breathomics as they have revealed a reproducible pattern of ventilatory variations and respiratory homeostasis in endogenous VOC exhalations.

## Introduction

Human breath is a timeless source of in vivo metabolic, physiological/pathophysiological information, especially for lung conditions^1–3^. Practice in pulmonology and breathomics have gradually encountered ubiquitous confounders; among which subject’s own respiratory physiology associated variations (intra-/inter-individual) are crucial and evident^4–6^. As breathomics suffered from insufficient fundamental knowledge on the effects of ventilatory variations, despite many efforts, pilot findings differed during independent validations. Thereby, breathomics couldn’t attain routine practice. Studies have witnessed fluctuations in cardiorespiratory variability (e.g. varying vagal tone, nonlinear cardiopulmonary coupling etc.) under voluntary and/or involuntary breathing^7–9^. Simple changes in ventilation/hemodynamics take place due to changes in breathing patterns^10,11^, oral/nasal routes^11^ and postures^12^. Such changes immediately affect bronchopulmonary gas-exchange, which alters exhalation of end-tidal CO_2_ and volatile organic compounds (VOCs).

When alveolar sampling^13^ is combined with real-time mass spectrometry e.g. Proton Transfer Reaction Time-of-Flight Mass Spectrometry (PTR-ToF–MS)^14^, breath-resolved evaluation of physiology driven changes and variations in VOC exhalations are possible. Previously, we employed continuous VOC profiling to track minute but significant physiological fluctuations under varying respiratory flow, -exhalation kinetics, -forced expiration^15^ and under increased upper-airway resistances^16^. Thus, while VOC profiling enabled us to model complex physiological and metabolic variability^17^, we realised that any normal but unsupervised physiological effects during sampling may override the actual pathophysiological impression in obtained data. This may lead us to clinical misinterpretation of results. To overcome such hurdle, a comprehensive understanding of normal ventilatory variations and its play in bronchopulmonary gas- and VOC exchange is inevitable. We have learned that in order to minimize/discriminate physiological variations, one must maintain certain ventilatory attributes (i.e. sampling related) in any clinical study concerning breathomics.

Evidences showed that the control of breathing is mainly automatic and its regulation is keen to autonomic functions^18^. Thus, spontaneously breathing healthy subjects start to hyperventilate once they are asked to breathe normally^19^ and such effects are assumed to appear from the autonomic nervous regulation of the respiratory centre in human brain^20^. Therefore, those effects will certainly reflect in exhaled alveolar concentrations of VOCs, whose exhalation are partially or directly dependent on minute ventilation and/or on CO_2_ exhalation^11^. Ventilatory variability is known to cause random disturbances in arterial CO_2_ pressure levels, cardiac output and pulse pressure in human^21,22^. Despite its vast clinical scope and applicability^23–25^, the influences of breath-resolved ventilatory variations (i.e. during unsynchronised/involuntary and synchronised/voluntary respiration) onto exhaled end-tidal CO_2_ and VOC exhalation have not yet been investigated in minute detail. Undoubtedly, paced (i.e. voluntary) and spontaneous (i.e. involuntary) rhythms have vast clinical and experimental importance in respiratory medicine and breathomics^26–28^. Therefore, real-time evaluation of such effects is crucial in order to understand the basic behaviour of respiratory fluctuations under varying respiratory rhythms. Here, we applied real-time high-resolution mass-spectrometry in combination with breath-resolved spirometry and capnometry to investigate effects of ventilatory variations (under varying respiratory rhythms) onto the exhaled breath components in healthy conscious humans. We focused mainly on the relative changes and variations in main determinant of alveolar ventilation, bronchopulmonary gas-exchange and most abundant endogenous VOC exhalations. We have addressed the following questions in detail:

- What are the immediate effects of varying respiratory rhythms onto exhaled breath compositions?
- Can certain respiratory rhythm reduce and reproduce physiological variations?
- Can we model real-time ventilatory variations in minute ventilation and pET-CO_2_ via breath-resolved VOC exhalation?

## Results

**Figure 1:**
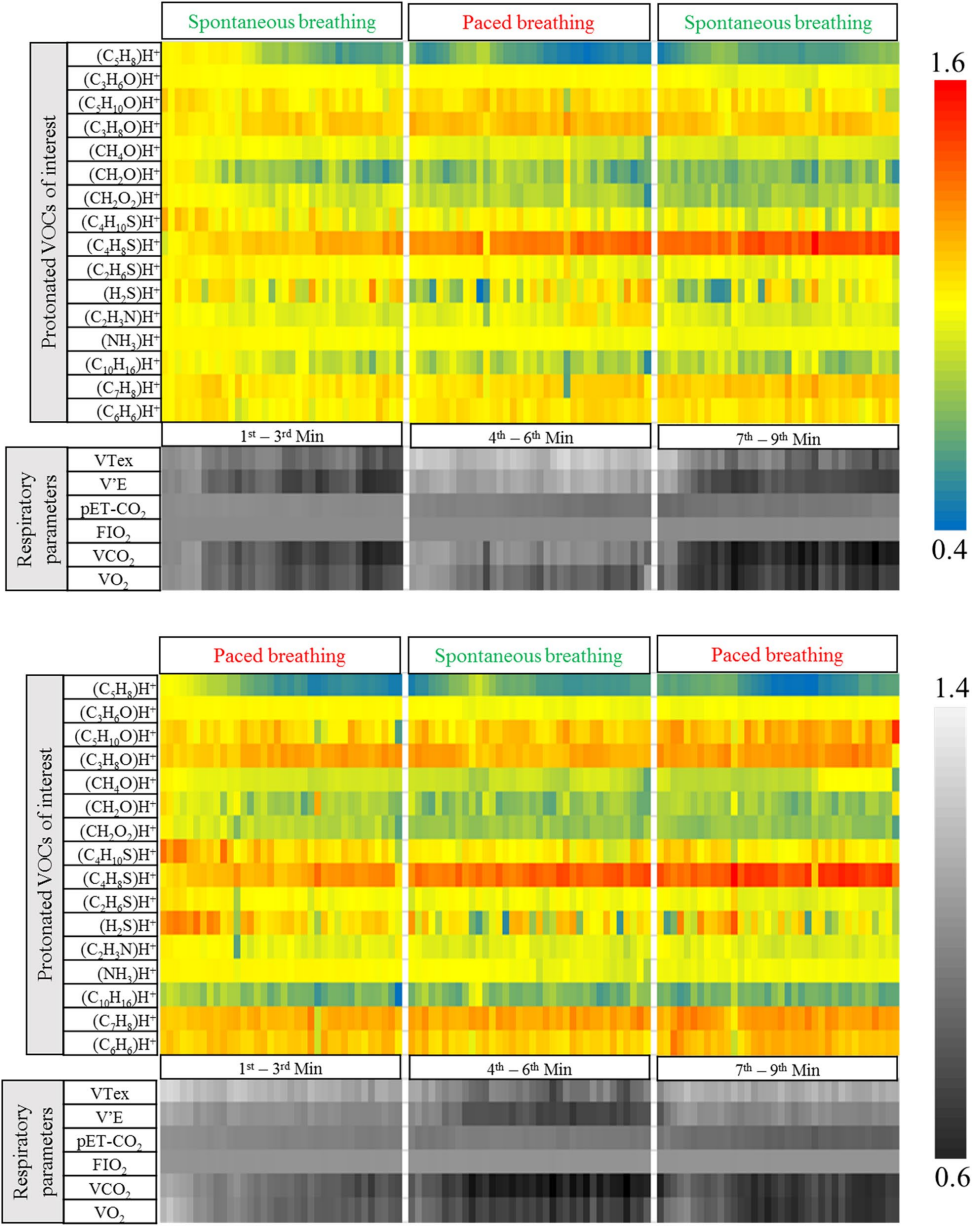
Relative changes in normalised mean alveolar concentrations of selected substances and respiratory parameters from 25 healthy subjects during administration of different respiratory rhythms: Setup-1 (top); Setup-2 (bottom). VOCs were tentatively identified based on their m/z. Respiratory rhythms were changed every 3 min. VOC data (from both setups) were normalised on to corresponding values in the second exhalation of setup-1. Respiratory parameters were normalised in the same way. VTex = expiratory tidal volume, pET-CO_2_ = end-tidal partial pressure of carbon dioxide, V′E = minute ventilation, FIO_2_ = fraction of inspiratory oxygen, V’CO_2_ = carbon dioxide production, V’O_2_ = oxygen consumption. For VOCs, changes in colours from red to blue symbolise relative changes from higher to lower concentrations and vice versa. Similarly, for respiratory parameters, changes in colours from light grey to black represent relative changes from higher to lower values and vice versa.

The heat-maps in Fig. 1 represent the non-quantitative expressions of relative changes in exhaled alveolar concentrations of selected VOCs and respiratory parameters throughout the study. In this study we measured hundreds of VOCs in real time by PTR-ToF. To reduce confounding effects, only compounds with expiratory abundances well above the inspiratory abundance (Supplementary Fig. S1 online) were selected for further analysis. Out of those, we selected 16 compounds reflecting a broad spectrum of chemical classes and physico-chemical properties to address substance-specific effects. These substances reflect important aspects for clinical breathomics such as different origins (endogenous and blood borne, oral cavity, previous exposure) and different dependencies on physiology and metabolism. Switching respiratory rhythm caused immediate changes in many measured parameters within seconds.

In order to frame ventilatory variations under varying respiratory rhythms via VOC exhalation, we emphasised two most abundant and bloodborne VOCs in human breath. Acetone and isoprene have different physico-chemical properties (e.g. aqueous solubility, volatility, blood-gas partition coefficient etc.) and are therefore reflecting different effects of exhalation kinetics and respiratory physiology such as alveolar and bronchial gas-exchange, dependency on ventilation, haemodynamic and compartmental distributions. In conventional clinical (routine-) practice in pulmonology and respiratory medicine pET-CO_2_ and minute ventilation are used as principal determinants to monitor/control bronhco-pulmonary gas-exchange physiology and associated ventilatory parameters and derivatives. As acetone and isoprene closely mirror (positively and/or negatively) many behaviours of those two respiratory parameters upon varying physiological/pathophysiological conditions in real-time, we selected these two VOCs in order to address effects of respiratory rhythms on ventilatory variations. Breath-resolved ventilatory variations of VOCs from various substance classes and origin are presented in Supplementary Fig S2 online. Statistical validation and reproducibility in breath-resolved ventilatory variations in 67 measured volatile masses (selected from Supplementary Fig. S1 online, depending on their relatively higher abundances in exhalation than in inspiratory room-air) are presented in Supplementary Table S2 online. While compared to the last minute (M3) of the initial spontaneous breathing form setup-1, the median range of ventilatory variations in measured VOCs decreased significantly (p-values ≤ 0.005) by ~ 5–15% at the last minute (M9) of the final spontaneous rhythm of setup-1 and such reduction was reproduced during the last minute (M6) of the spontaneous rhythm in setup-2. RM-ANOVA results of all pairwise multiple comparisons are presented in Supplementary Table S3 online.

**Figure 2:**
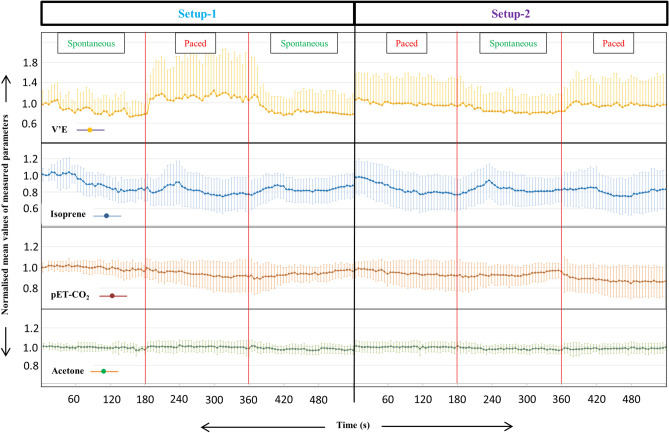
Continuous breath-resolved changes in normalised mean alveolar concentrations of endogenous isoprene and acetone along with minute ventilation and pET-CO_2_ from all participants in both setups. X-axis represents measurement time in both setups. Y-axis represents normalised (on to corresponding values in the second exhalation of setup-1 for each subject) mean values of measured parameters. Respiratory rhythms were changed every 3 min and such time points are divided via red lines.

Breath-resolved time profiles of V’E, pET-CO_2_, isoprene and acetone are presented in Fig. 2. Haemodynamic parameters (e.g. cardiac output) did not change considerably and therefore are excluded from results and discussion.

**Figure 3:**
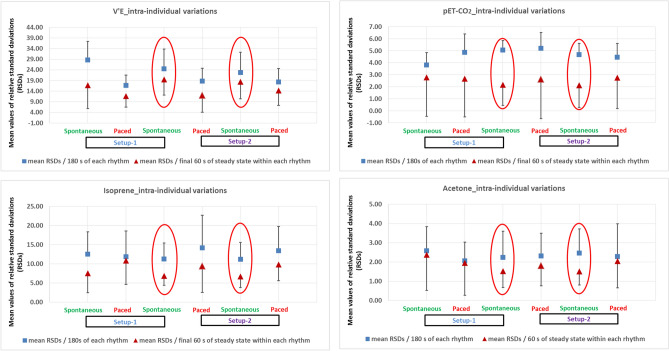
Comparison of differences in intra-individual variations of respiratory parameters, -isoprene and acetone within both setups. X-axis represents spontaneous respiratory rhythms from both setups. Y-axis represents mean values of relative standard deviations (i.e. RSDs in %) of measured parameters. RSDs over 180 s of all rhythms were compared to each other. Comparisons were also performed over final 60 s (steady state) of each rhythm. V’E and pET-CO_2_ values were compared in the same way. Statistical significances were tested by means of repeated measurement-ANOVA on ranks (*p*-value ≤ 0.005). From all pairwise-multiple comparisons, statistically indifferent (i.e. with no significant differences and thereby, reproducible) respiratory rhythms are marked with red-coloured ovals.

Relative changes (in %) in alveolar concentrations (with corresponding *p*-values) of most abundant endogenous VOCs and relevant respiratory parameters are listed in Table 1. Intra-individual variations (mean RSDs in %) over 180 s and over final 60 s (steady state) of each rhythm are presented in Fig. 3.

**Table 1:** Statistical significance of observed changes in exhaled alveolar abundance of isoprene, acetone, pET-CO_2_ and respiratory parameters.

(VOC)H + & respiratory parameters	Mean RSDs (%)		Significance (P ≤ 0.005)/median/180 s	Median changes (%) of normalised mean/180 s	Respiratory rhythms	Study setup	Respiratory rhythms	Median changes (%) of normalised mean/180 s	Significance (P ≤ 0.005)/median/180 s	Mean RSDs (%)		(VOC)H + & respiratory parameters
	/final 60 s (rteady state)	/180 s								/180 s	/final 60 s (steady state)	
**(Isoprene)H + 69.06989 (g/mol)**	7.57	12.50	< 0.005	7.61	Spontaneous	**Setup-1**	Spontaneous	3.86	< 0.005	28.64	16.83	**Minute Ventilation [L/min]**
	10.86	11.86	< 0.001	-3.42	Paced		Paced	38.75	< 0.001	16.68	11.71	
	**6.86**	11.26	N/A	0	**Spontaneous**		**Spontaneous**	0	N/A	24.46	**19.55**	
	9.43	14.16	< 0.001	-2.11	Paced	**Setup-2**	Paced	20.73	< 0.001	18.76	11.98	
	**6.72**	11.22	> 0.005	-0.84	Spontaneous		Spontaneous	1.98	> 0.005	22.82	**18.42**	
	9.84	13.45	< 0.005	-1.83	Paced		Paced	17.17	< 0.001	18.38	14.34	
**(Acetone)H + 59.04914 (g/mol)**	2.38	2.58	< 0.005	1.78	Spontaneous	**Setup-1**	Spontaneous	8.59	< 0.001	3.82	2.78	**pET-CO**_**2**_** [kPa]**
	1.95	2.05	< 0.001	2.58	Paced		Paced	1.36	> 0.005	4.88	2.68	
	**1.53**	2.23	N/A	0	**Spontaneous**		**Spontaneous**	0	N/A	5.05	**2.17**	
	1.80	2.31	< 0.005	2.28	Paced	**Setup-2**	Paced	2.24	< 0.005	5.21	2.62	
	**1.51**	2.46	> 0.005	0.20	Spontaneous		Spontaneous	-0.37	> 0.005	4.67	**2.13**	
	2.05	2.29	> 0.005	0.60	Paced		Paced	-5.58	< 0.001	4.46	2.76	
**V’O**_**2**_** (ml/min)**	17.35	27.23	< 0.001	12.21	Spontaneous	**Setup-1**	Spontaneous	15.36	< 0.001	28.24	17.20	**V’CO**_**2**_** (ml/min)**
	13.96	17.24	< 0.001	16.85	Paced		Paced	41.13	< 0.001	16.80	13.61	
	**18.99**	23.68	N/A	0	**Spontaneous**		**Spontaneous**	0	N/A	26.79	**18.91**	
	12.22	23.95	< 0.001	15.29	Paced	**Setup-2**	Paced	24.57	< 0.001	20.99	12.24	
	**19.13**	20.79	> 0.005	5.19	Spontaneous		Spontaneous	2.78	> 0.005	22.37	**19.11**	
	18.12	19.80	> 0.005	3.85	Paced		Paced	10.31	< 0.001	19.58	18.20	

Relative changes in normalized mean values and relative standard deviations (mean RSDs i.e. calculated over the 180 s and over final 60 s of measurement over each respiratory rhythm) of measured parameters, respectively. Exhaled VOC concentrations, pET-CO_2_, V’O_2_, V’CO_2_ and minute ventilation from same respiratory rhythms (over 180 s) were compared within- and between two setups. Statistical significances were tested by means of repeated measurement-ANOVA on ranks (*p*-value ≤ 0.005). From all pairwise-multiple comparisons, we selected those referring to the final 180 s of spontaneous (i.e. presented in bold fonts) rhythm from setup-1. Significance test was not applicable (N/A) to the reference point itself and thus the median changes are assigned to zero (0). Changes with a resulting *p*-value ≤ 0.005 were regarded as significant. The reproducable RSD values at the steady states of the spontaneous rhythms from setup-1 and setup-2 are also presented in bold fonts.

**Figure 4:**
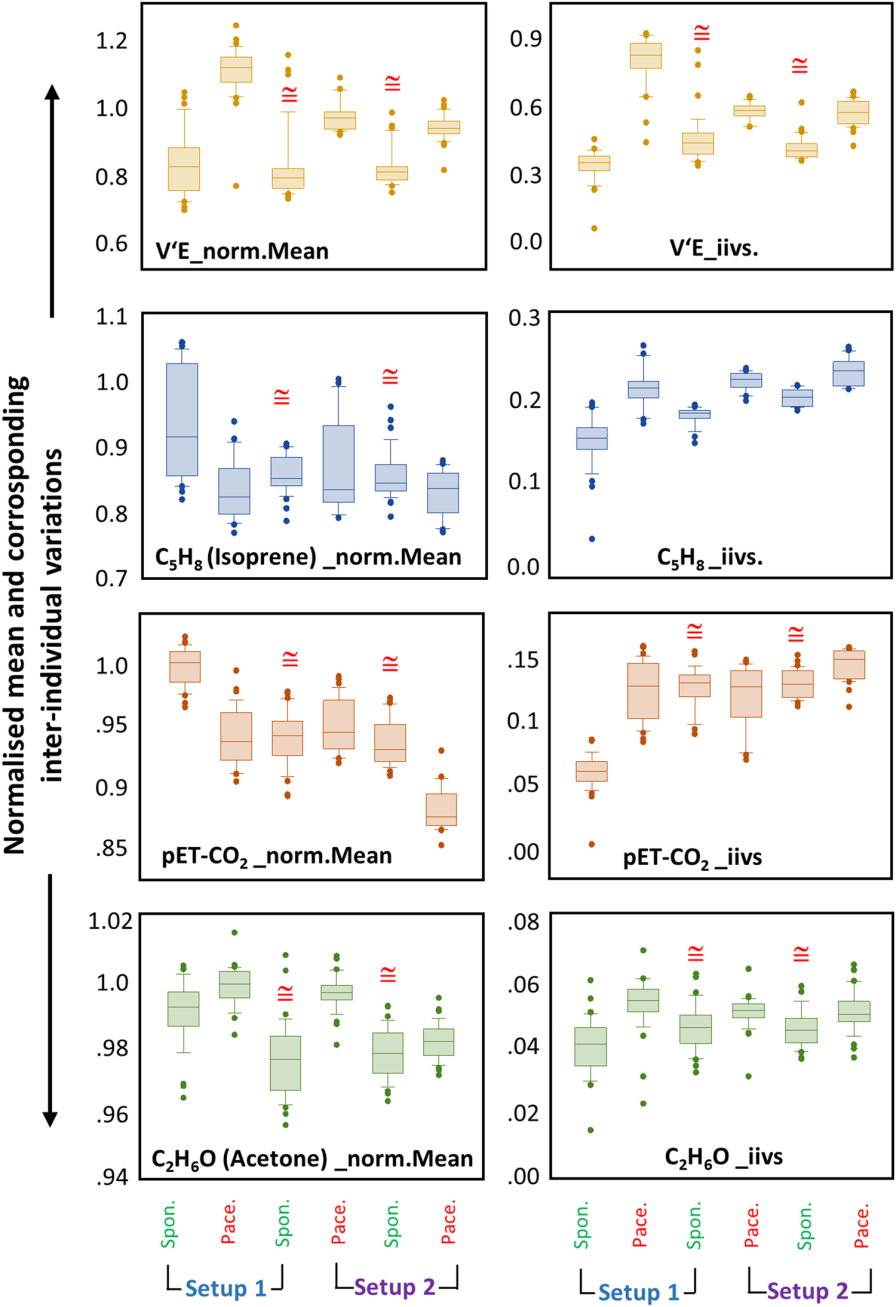
Comparisons of exhaled alveolar concentrations and corresponding inter-individual variations of endogenous isoprene, -acetone and respiratory parameters. X-axis represents two study setups with three respiratory rhythms in each. Y-axis represents normalised mean (_norm.Mean) values and inter-individual variations (_iivs.) of measured parameters, respectively. Exhaled VOC concentrations and intra-individual variations from same respiratory rhythm (over 180 s) were compared within- and between two setups. V’E and pET-CO_2_ values were compared in the same way. Statistical significances were tested by means of repeated measurement-ANOVA on ranks (*p*-value ≤ 0.005). From all pairwise-multiple comparisons, statistically indifferent (i.e. with no significant differences and thereby, reproducible) rhythms are marked with red-coloured ‘ ≅ ’ for respiratory phases.

**Figure 5:**
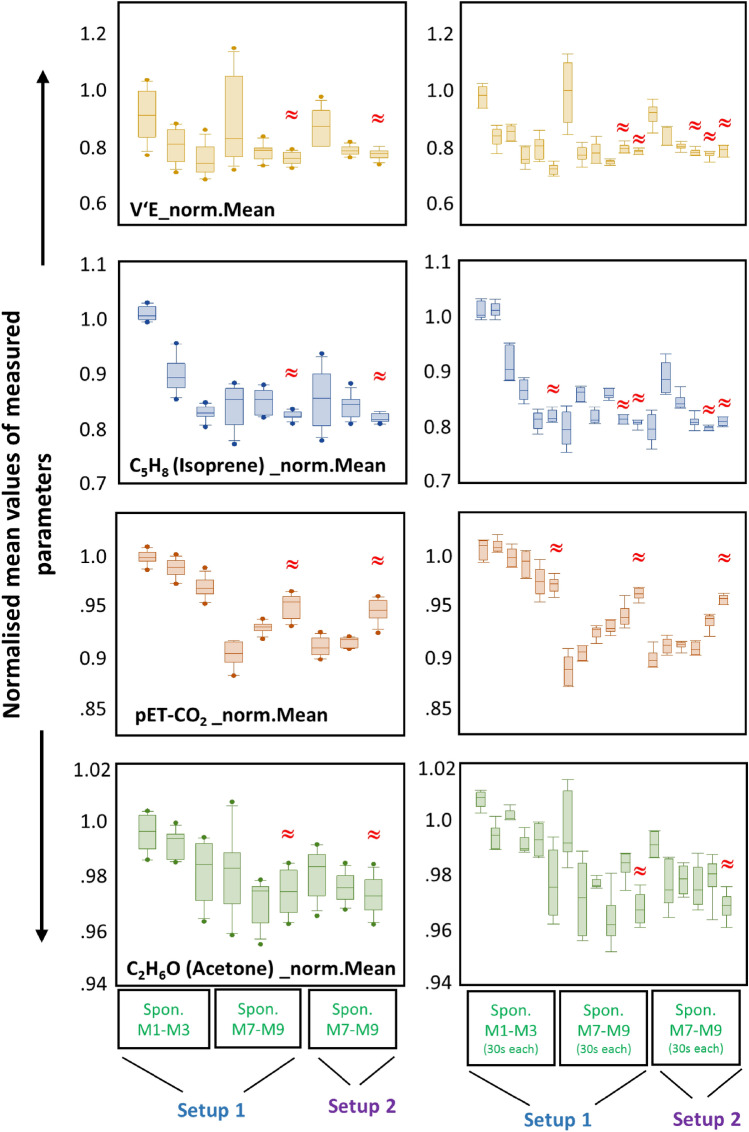
Comparison of differences in exhaled alveolar concentrations of endogenous isoprene, -acetone and respiratory parameters during spontaneous breathing in both setups. X-axis represents spontaneous respiratory rhythms from both setups. Y-axis represents normalised mean (_norm.Mean) values of measured parameters, respectively. Exhaled VOC concentrations from spontaneous rhythms were compared. Comparisons were performed over 60 s (presented on reader’s left) and over 30 s (presented on reader’s right) of intervals. V’E and pET-CO_2_ values were compared in the same way. Statistical significances were tested by means of repeated measurement-ANOVA on ranks (*p*-value ≤ 0.005). From all pairwise-multiple comparisons, statistically indifferent (i.e. with no significant differences and thereby, reproducible) spontaneous breathing intervals are marked with red-coloured ‘≈’.

Comparison of differences between normalized mean (over 180 s) and corresponding inter-individual variations in those variables, which are obtained from each respiratory rhythm (in setup-1 and -2) are represented as boxplots in Fig. 4. From all pairwise comparisons between same rhythms, the following outcomes are most crucial:

- Measured parameters from the initial spontaneous rhythm (i.e. 1st–3rd minute) from setup-1 was significantly different than the same from setup-1 (i.e. 7th–9th min) and setup-2 (i.e. 4th–6th min).
- While preceded by a paced rhythm, measured breath components from a subsequent spontaneous rhythm in both setups were reproducible. Therefore, no differences were observed in any of the above indicated parameters from the final spontaneous rhythm (i.e. 7th–9th min) of setup-1 with those from spontaneous rhythm (i.e. 4th–6th min) of setup-2.
- The above fact was true for both intra- and inter-individual variations.
- Above mentioned instances were not observed in case of paced rhythms.
  Comparison of differences in minute ventilation, end-tidal CO_2_, isoprene and acetone concentrations within the three spontaneous rhythms from both setups are presented as boxplots in Fig. 5. Comparisons were performed over 60 s (presented on reader’s left) and over 30 s (presented on reader’s right) of intervals. From all pairwise comparisons, the following results are remarkable:
- In both setups, only while preceded by paced breathing, the interquartile range of distribution (i.e. data variance in spontaneous phase) along with both inter-individual (i.e. percentile) and intra-individual variations (i.e. in %) in measured parameters were decreased significantly and most pronouncedly during the final minute (i.e. especially during the last 30–40 s of the 3rd min) of such spontaneous phases.
- Those two inter-setup spontaneous phases were statistically indifferent to each other and both were even significantly different than that of the initial 3 min of spontaneous breathing (i.e. not subsequent to a paced rhythm) from setup-1.
- Breath samples (exhaled alveolar concentrations and variations) from such spontaneous phases (i.e. preceded by a paced rhythm) were reproducible in both setups.

### Models of real-time ventilatory variations

**Figure 6:**
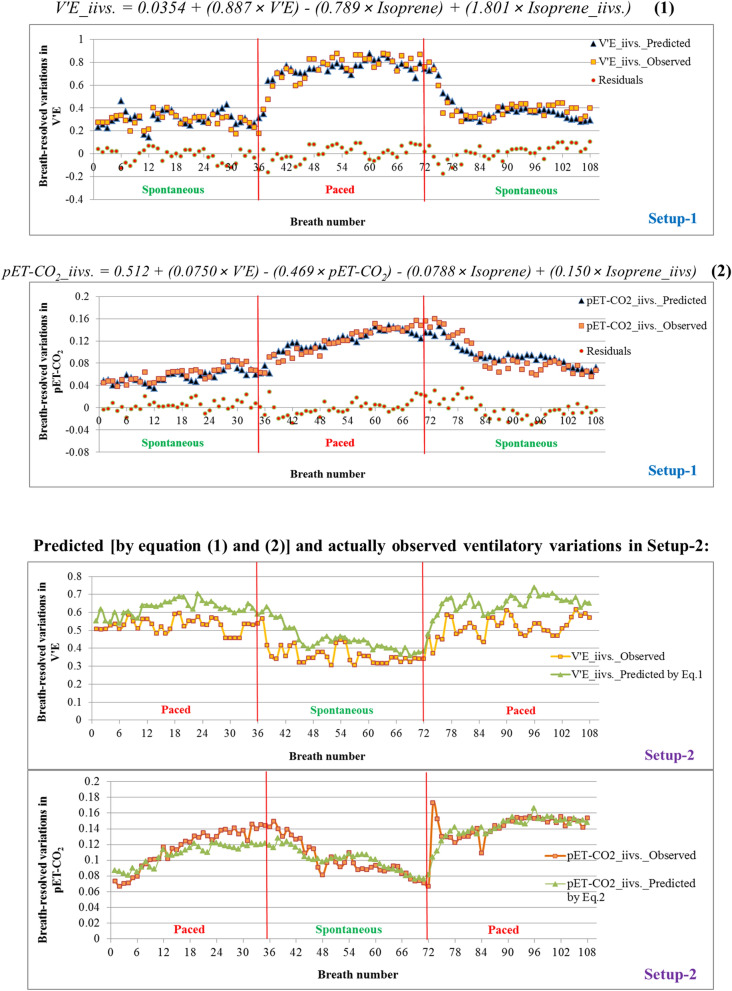
Regression models [Eqs. (1) and (2)] from Setup-1 are predicting breath-resolved ventilatory variations: Equations are presented along with corresponding prediction scores and residual plots. Predicted [by Eqs. (1) and (2)] and actually observed ventilatory variations in Setup-2: Measured data from setup-2 were applied onto Eqs. (1) and (2) in order to predict breath-resolved variations in V’E and pET-CO_2_, respectively. X-axis represents breath numbers. Y-axis represents actually observed values and predicted values of breath-resolved variations in V’E and pET-CO_2_. The Kolmogorov–Smirnov test showed excellent agreement between the independently predicted ventilatory variations in setup-2 and the actual measures.

Breath-resolved ventilatory variations in spirometric and capnometric parameters were closely related to the exhaled alveolar concentrations and corresponding inter-individual variations of endogenous and blood borne isoprene. Here, multiple linear regressions models [Eqs. (1) and (2)] are predicting breath-resolved ventilatory variations in minute ventilation and pET-CO_2_ via the exhalations of isoprene during varying respiratory rhythms. Both equations were derived from Setup-1 and presented along with corresponding prediction score plot and residual plot in Fig. 6 and corresponding summary output e.g. R square, statistical significance, *p*-values etc. are presented at Supplementary Table S1 online.

### Regression equations

In order to realize the applicability and reliability of Eq. (1) and (2) [derived from Setup-1] for predicting ventilatory variations, we tested those in the independent setup-2. Here, the measured/analysed data from setup-2 were applied on those equations to predict breath-resolved ventilatory variations in V’E and pET-CO_2_. Observed scores and predicted scores are presented in Fig. 6.

1 $$V^{\prime}E\_iivs. \, = \, 0.0354 + \left( {0.887 \times V^{\prime}E} \right) - \left( {0.789 \times Isoprene} \right) + \left( {1.801 \, \times Isoprene\_iivs.} \right)$$

2 $$pET - CO_{2} \_iivs. = 0.512 + \left( {0.0750 \times V^{\prime}E} \right) - \left( {0.469 \times pET - CO_{2} } \right) - \left( {0.0788 \times Isoprene} \right) + \left( {0.150 \times Isoprene\_iivs.} \right)$$

The Kolmogorov–Smirnov test showed excellent agreement between the independently predicted ventilatory variations in setup-2 (Fig. 6) and the actual measures. Throughout setup-2, no significant differences (*p*-value = 0.441) in breath-resolved ventilatory variations for pET-CO_2_ is observed. Similar insignificance (*p*-value = 0.131) in variations for V’E is observed exclusively during the final steady state of spontaneous breathing phase.

## Discussion

In order to bridge a crucial knowledge gap to implement breathomics in routine respiratory medicine, we investigated the immediate effects of ventilatory variations on exhaled breath composition, under varying respiratory rhythms. Unprecedented reproducibility in the measured variables is obtained via a combination of respiratory rhythms i.e. reliable for sampling and could increase our perseverance of true pathophysiological expressions in exhaled matrices. Ventilatory variations were modelled via alveolar VOC exhalation i.e. validated independently with high precision. Our findings could translate breathomics towards the domain of applied respiratory medicine.

### Respiratory parameters

In practice, respiratory rate of 10–12 breaths/min with breath-resolved I:E ratios of ~ 1:2 are regarded as normal spontaneous breathing at rest in healthy adults^29^. Nevertheless, healthy adults with relatively higher lung capacity often have relatively lower respiratory rates but the same I:E ratio. During paced breathing in both setups, all volunteers with varying and increased tidal volume had to breathe ~ 12 times a minute where the mean I:E ratio was changed to ~ 1:1.75. Thus, V’E increased significantly under metronome controlled fixed respiratory rate. In this case, corresponding ventilatory variations (inter-individual) also increased pronouncedly due to varying physiological effects. To maintain the blood-gas homeostasis, the respiratory centre mediate an automatic and involuntary control of breathing^18,30^. Thus, physiological effects from voluntary control (i.e. momentary and not automatic) via conscious factor e.g. under paced breathing are mandated to neutralize eventually^18^. Once switched to spontaneous breathing, V’E returned to normal range and corresponding ventilatory variations lowered significantly as soon as the precedent physiological effects were normalized^31^. As the autonomic regulation and response to ventilatory control is programmed as somatic motor functions^18^, a defined magnitude of induced hyperventilation is likely to normalize at a reproducible extent within a reproducible time frame. As in this phase *p*CO_2_ is lower than normal, there is no room for further involuntary hyperventilation. The aforementioned instances explain the unprecedented inter-setup reproducibility of breath compositions during spontaneous rhythms (viz. preceded by paced rhythm).

In principle, in spontaneously breathing (i.e. with RR = 10–12/min and I:E ratio of 1:2) awake young and healthy adults, mean ± SD of expiratory tidal volume (VTex) range ~ 0.5 ± 0.2 (L) or ~ 7 mL/kg of body weight in normal condition. During breath sampling one must maintain such normal range in order to overcome physiological influence. In practice this is utterly difficult. In our setup, at the 1st minute of the initial spontaneous breathing (Setup-1) the mean ± SD of expiratory tidal volume was ~ 0.55 ± 0.2 (L) and during third minute was ~ 0.6 ± 0.2 (L). This happened as subjects started to relatively hyperventilate (i.e. physiological) due to overtaking conscious voluntary control of breathing (as discussed in the manuscript). Interestingly, while preceded by paced breathing, the VTex range was ~ 0.65 ± 0.2 (L) at the beginning of spontaneous rhythm and gradually at the third minute, VTex had reached and stayed at 0.5 ± 0.2 (L), which denotes to normal conditions.

Such physiological effects were not contributed randomly by any/some subjects as the same behaviour of normalized patterns were observed while subjects were treated as his/her (own) control. Intra-individual variations (mean RSDs) of V’E decreased significantly during paced rhythms due to the fixed RR.

### Exhaled end-tidal CO_2_

pET-CO_2_ is non-invasive substitute of arterial CO_2_ pressure (*Pa*CO_2_) as the difference has been measured to be only 1–2 mmHg in healthy subjects. The normal range of pET-CO_2_ (i.e. measured by tidal volume) ranges between 35 and 45 mmHg in healthy adult humans. A deviation of ± 3.5–5 mmHg is regarded as normal variation^32,33^, which represents the physiological range of relative hyperventilation in healthy adults. During the initial spontaneous rhythm of setup-1, normalized median of pET-CO_2_ values decreased gradually (by ~ 5–7%) and reached a steady state during the final minute, which supports the fact that autonomic responses (i.e. instruction driven and not automatic) to respiratory centre let conscious subjects hyperventilate, if they are asked to breathe normally.

In normal sitting posture at rest, pET-CO_2_ exhalation is negatively correlated to minute ventilation as an elevated V’E increases V’CO_2_ and thereby decreases *Pa*CO_2_ and pET-CO_2_ (Table 1). Thus, pET-CO_2_ decreased significantly at paced rhythms as elevated V’E induced relative hyperventilation (*Correlation coefficient:* − 0.729*, p-value* < 0.005). Consequently, once switched to spontaneous breathing, CO_2_ concentrations gradually attained the steady state (during final minute) with significantly lowered ventilatory variations.

Thereby, we have expanded the conventional concept of physiological hyperventilation by demonstrating that it takes place, not only while subjects are told to breathe normally but also if they are instructed to maintain a normal respiratory rate. Therefore, any arbitrary application of either spontaneous or paced breathing cannot be justified for breath sampling as random ventilatory effects and variations may override the true pathophysiological impression of data and mislead clinical interpretations.

### Exhalation of endogenous acetone

Having its potential origin from glycolysis and lipolysis^34^ acetone is the most abundant VOC in human exhalation. Acetone has aqueous miscibility, moderate volatility and high rate of compartmental distribution. Thus, the substance remained even independent of pronounced changes in hemodynamics^12^. In our setups, alveolar acetone concentrations and corresponding variations closely mirrored V’E and the exhaled concentrations were slightly (but significantly) higher during paced breathing. Here, a unavoidable increase in respiratory flow (and altered alveolar slope) due to increased V’E might lead to extra-alveolar exchange of highly soluble compounds like acetone^15,35–37^. In accordance with the MIGET theory^38,39^, such reproducibility could be assigned to acetone’s good aqueous solubility as of CO_2_.

### Exhalation of endogenous isoprene

Dating its anticipated origin from cholesterol biosynthesis^40^ isoprene is the second most abundant endogenous VOC in breath. Due to having low aqueous solubility but high volatility, alveolar isoprene mirrors the pulmonary ventilation-perfusion effects^10,12^. Conscious and unconscious muscle movements affect isoprene exhalation^41,42^. Previously we observed changes in alveolar isoprene concentration under varying respiratory muscle activity^16^. In this setup, switching between respiratory rhythms resulted with immediate increase in isoprene concentrations within the first minute and was normalised to baseline within the next minute. A simultaneous rise in measured V’O_2_ indicates a positive change in respiratory muscle work load under conscious control of the respiratory centre^43^, due to voluntary switching of rhythms. Thus, the increase in isoprene exhalation can be attributed to its better washout from respiratory muscles. On the other hand, the unprecedented reproducibility in isoprene exhalation during spontaneous rhythms (viz. preceded by a paced breathing) can be attributed to the same effects of a defined magnitude of hyperventilation on *p*CO_2_ i.e. explained earlier.

### Exhalation of other VOCs

Respiratory rhythms driven changes in concentrations of many other VOCs remained unrelated to their ventilatory variations. Despite varying changes in concentrations of these VOCs, significant and reproducible decrease in variations took place during the final steady state of spontaneous rhythms (viz. preceded by a paced breathing) in all measured masses. Due to the analytical limitation of PTR-MS, changes in ammonia exhalation could not be measured with sufficient precision^44^. As all subjects were not exposed to exogenous compounds like acetonitrile (originated via smoking), benzene and toluene (derived from pre-exposure)^45^ we could not draw any systemic relevance. Oral cavity originated compounds such as hydrogen sulphide, allyl-methyl-sulphide, propionic acid and ethanol (attributed to bacterial emission or dietary remains) showed wash out effects without any considerable relation to ventilatory variations^46^. Dimethyl sulphide and methyl-propyl-sulphide concentrations remained constant due to their potential origin from gut and nasal cavity bacteria, respectively^11^. Formaldehyde was also present in inspiratory room air and therefore, its exhalation was dependent on the semi-exogenous origin. Pentanaldehyde could not be quantified in many participants. Formic acid exhalation remained constant as it was originated from the disinfectant, used to sterilize the spirometric flow-volume sensor.

### Contribution towards clinical breathomics

Realizing the importance of changes in VOC concentrations rather than the anticipated existence of any unique/event-specific volatile biomarker in clinical breathomics, standardization of breath sampling has come into spotlight^47,48^. Due to exhaustive list of confounders and regular irregularity of sampling- and analytical standards in the field of breathomics^49^, validation/reproducibility of results are compromised^50^. Not surprisingly, even less pronounced changes and variations in endogenous VOC concentrations (than i.e. observed in this study) are misinterpreted as disease biomarker in cross-sectional pilot studies, which could not be reproduced further. During sampling, normal (but complex) variability of respiratory physiology often screens the actual (rather minute) expressions of pathological- or metabolic condition in the VOC data^11,12^. Reductions of physiological effects (and corresponding variations) in obtained data are critical challenges that is faced since the inception of pulmonology and breathomics. In order to realize the anticipated future of breathomics applications in routine medical practice; subject’s own physiological dissimilitude must be diminished substantially. Here, we could significantly minimize such variations by applying a combination of respiratory rhythms in two independent setups. We hereby recommend the following protocol for breath sampling in awake conscious human: “After a minute (at least) of metronome controlled (with a normal RR of 10–12/min) paced rhythm switch to spontaneous rhythm and then start breath sampling from the third minute onward”.

Our above-recommended sampling protocol was able to reduce ventilatory variations in a wide-variety of VOCs (Supplementary Fig. S2 online). Previously we have reported that substances of similar origin and chemical class behaved alike during under physiological fluctuations e.g. breathing patterns, -routes, postures, prolonged exhalation or forced expiration, increased upper-airway resistances etc. As our candidate VOCs cover a wide range of physico-chemical properties (e.g. aqueous solubility, volatility etc.) these findings incorporate a broad range of volatiles (known and/or unknown). Herein, we have addressed the general need (irrespective of analytical techniques) to overcome the sampling related physiological confounders, which account for the standardization of clinical breath sampling.

### Translation of knowledge to pulmonology

As alveolar exchange of VOCs with low aqueous solubility and high volatility, can closely mirror many attributes of bronchopulmonary gas-exchange process^15,16^, we modelled continuous ventilatory variations in V’E and end-tidal CO_2_ exhalation via the exhalation of endogenous isoprene. Two models [Eqs. (1) and (2)] i.e. derived from setup-1, could predict breath-resolved ventilatory variations in the independent setup-2 with remarkable precision (Fig. 6). Our findings show that analysis of breath volatiles has the potential to provide valuable information beyond what can be deduced from *Sp*O_2_, pET-CO_2_ and conventional spirometry. Once translated into clinical implications, these equations may apprehend our fundamental and intelligible understanding of ventilatory variations in favour of its ubiquitous scope and applicability in respiratory medicine. Therefore, future research should apply these models into larger clinical trials in order to push the present state-of the-art domain beyond the cutting-edge expertise.

Our sampling protocol will help to see minute changes and variations in breath compositions beyond the everlasting physiological noise. While considering the current limitation, ventilatory variations (in respiratory parameters) were modelled upon the most abundant and endogenous VOCs, acetone and isoprene. Although, our sampling protocol driven reproducible reduction in VOC variations were validated upon a wide range of volatiles (Supplementary Table S2 online), those should be tested for any other substances, which may possibly appear in human breath. As for breath VOCs presented here, available data suggest that the same protocol might have an important influence on VOCs variability and reproducibility, thus should be generally applicable. However, substance specific attributes and validation of many other VOCs (that may appear in various patients) will be addressed via our protocol within subsequent future communications.

## Methods

### Healthy human subjects

Our study was conducted in accordance with the amended Declaration of Helsinki. Ethical approval (Approval number: A 2015-0008) from the Institutional Ethics Committee (University Medical Centre Rostock, Germany) and signed informed consent from 25 healthy adults were obtained. As inclusion criteria, subjects were aged between 18 and 50 years and were not suffering from any acute or chronic diseases or were not undertaking any special dietary supplement, medication or therapy. Subject’s demographic and spirometric data (lung function parameters) are listed in Table 2.

**Table 2:** Demographic data of healthy subjects.

Participant-ID	Age (years)	Sex	Height (cm)	Weight (Kg)	Smoker	BMI (Kg/m^2^)	FEV_1_ (%)	FEV_1_/FVC (ratio)
1	28	F	168	70	Yes	25	82.12	0.78
2	31	M	168	64	No	23	97.90	0.82
3	30	M	193	85	No	23	87.63	0.83
4	32	F	170	67	No	23	84.87	0.85
5	30	M	165	65	No	24	100.49	0.80
6	48	M	186	78	No	22	88.70	0.76
7	48	F	165	65	Yes	24	91.60	0.81
8	28	M	183	75	No	22	88.17	0.82
9	34	M	184	72	No	21	87.89	0.87
10	45	F	168	60	Yes	21	81.61	0.77
11	27	F	175	63	No	21	96.89	0.76
12	49	M	195	102	No	27	94.15	0.81
13	26	F	167	71	No	26	88.66	0.81
14	30	F	171	80	Yes	27	91.33	0.80
15	30	M	176	87	No	28	100.79	0.85
16	26	M	180	92	No	28	100.57	0.86
17	25	F	155	48	No	20	99.23	0.83
18	22	M	186	84	No	25	98.30	0.85
19	27	F	163	65	No	24	99.23	0.95
20	23	M	178	75	No	24	101.19	0.93
21	28	F	170	63	No	22	99.30	0.86
22	32	M	172	68	No	23	100.29	0.80
23	29	M	181	89	Yes	27	88.15	0.77
24	29	F	165	65	No	24	99.40	0.89
25	32	F	173	75	No	25	86.63	0.84

Participant’s age, sex, height, body weight, smoking habit, body mass index (BMI), Forced expiratory volume in one second (FEV_1_ performance in %) and FEV_1_/FVC ratio (i.e. the Tiffeneau-Pinelli index) are listed.

### Study setup and protocol

**Figure 7:**
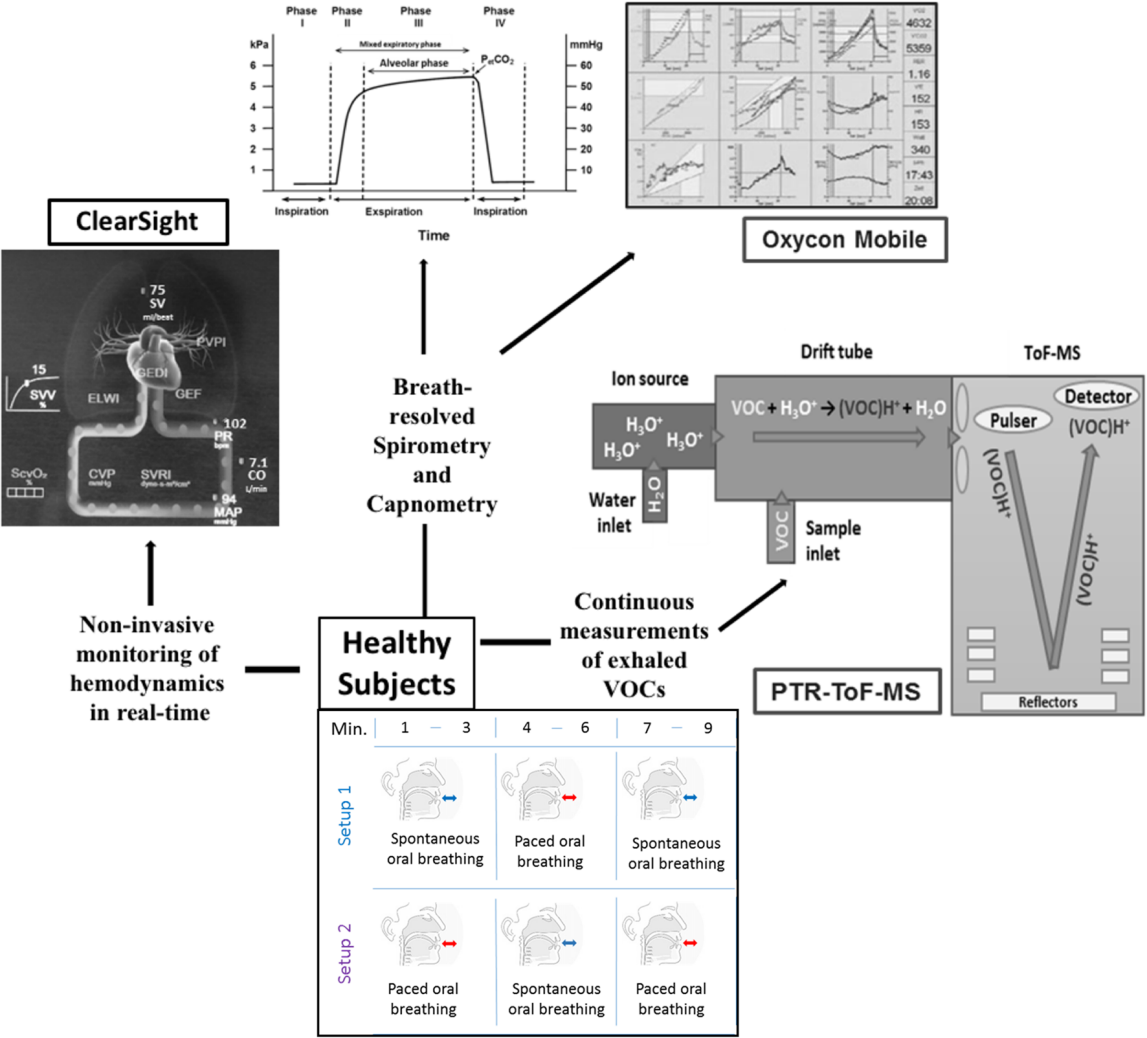
A scheme of study setup-1 and setup-2. This indicates the switching of respiratory rhythms over time along with all analytical and clinical instrumentations.

In this study, three devices respectively for real-time measurements of breath VOCs, respiratory- (i.e. via breath-resolved spirometry and capnometry) and hemodynamic (i.e. non-invasively) parameters were synchronised together and we initiated data acquisition in parallel. Please see Fig. 7.

All volunteers took part in two independent setups. Subject maintained a normal sitting position and performed oral breathing (inhalation and exhalation via mouth only) via custom made Teflon-mouthpiece of 2.5 cm diameter^16^. Volunteers rested by sitting for at least 10 min before participating. A minimum interval of 30 min was used between the two setups in order to neutralize all effects and changes from setup-1. The transfer-line of PTR-ToF–MS, a spirometric flow-volume sensor and a capnometric sampling tube was connected to the mouthpiece in order to continuously record VOC concentrations, spirometric- and capnometric parameters, respectively. We reused mouthpieces for each participant after sterilisation. In order to avoid any additional and unsupervised nervous stimulus^19^, we did not use nose clips in these setups. Omitting nose clip does not prevent all influencing perturbations. Our aim was to overcome confounding effects as much as possible. All healthy subjects were instructed to inhale and exhale only via mouth and we applied breath-by-breath spirometry for the assessment of tidal volume, minute ventilation. Our real-time observations were in accordance to our previous investigation on the real-time effects of oral and/or nasal routes of breathing onto exhaled breath profiles^11^.

In setup-1, volunteers started spontaneous breathing and after 3 min switch to paced (with a fixed respiratory rate of 12 breaths/min by following a metronome beats) breathing. Finally, another 3 min of spontaneous breathing resulted in a total of 9 min of measurements.

In setup-2, subjects started paced breathing for 3 min, followed by 3 min of spontaneous breathing. Finally, another 3 min of paced breathing resulted in 9 min of measurements.

### PTR-ToF–MS measurements of breath VOCs

Breath VOCs were measured continuously via a PTR-ToF–MS 8000 (Ionicon Analytik GmbH, Innsbruck, Austria). We used pre-optimized experimental conditions^10,14^. Continuous side-stream mode of sampling via a 6 m long heated (at 75 °C) silco-steel transfer-line, which was connected to a sterile mouthpiece, subsequent to the spirometric flow-volume sensor. We used 20 ml/min of continuous sampling flow and the time resolution of the PTR-ToF–MS measurements was 200 ms. Thus, after every 200 ms we had a data point and on each data point hundreds of compounds were measured at their trace abundances (in both expiratory- and room air). The ion source current was set to 4 mA and the H_2_O flow was set to 6 ml/min. Drift tube temperature were set at 75 °C, voltage was 610 V and the pressure was 2.3 mbar. The resulting E/N ratio was 139 Td. After every minute a new data file was recorded automatically and the mass scale was recalibrated after each run (60 s). We used the following masses for mass calibration: 21.0226 (H_3_O^+^-Isotope), 29.9980 (NO^+^) and 59.049 (C_3_H_6_O).

### VOC data processing

VOCs were measured in counts per seconds (cps) and corresponding intensities were normalised onto primary ion (H_3_O^+^) counts. As PTR-MS continuously records both exhaled breath and inhaled room-air, we applied the ‘breath tracker’ algorithm (based on Matlab version 7.12.0.635, R2011a) to identify expiratory and inspiratory phases^10^. Here, we used acetone as the tracker mass as it is an endogenous substance, which has significantly higher signal intensity in expiration than in inhalation. As the high mass resolution of PTR-ToF–MS (4,000–5,000 Δm/m) can assign volatiles upon their measured mass and corresponding sum formula with high precision^11^, compound names are used while discussing results. VOCs are quantified via multi-component mixture of standard reference substances. Quantification process under adapted sample humidity (as in exhaled breath) using a liquid calibration unit (LCU, Ionicon Analytik GmbH, Innsbruck, Austria) is preestablished state-of-the-art^51^.

### Measurement of respiratory parameters

We used an Oxycon Mobile device (CareFusion GmbH, Hoechberg, Germany) for breath-resolved spirometry (i.e. breath-by-breath measurement of ventilatory parameters) and capnometry. Subsequent data analysis was performed via the inbuilt JLAB Software 5.3x (Version 02.00, CareFusion GmbH, Germany). This equipment fulfils the requirements for clinical and laboratory practice in accordance to the European Respiratory Society (ERS) and American Thoracic Society (ATS) standards.

The mechanism of action and functionality is already discussed in our previous studies^15,16^. In principle, the TripleV–SBx (flow-volume sensor—gas-sensor box) unit allows breath-resolved and intra-breath measurements of spirometric and capnometric data continuously in real-time. Prior to the inclusion of each participant, a reassessment of the ambient conditions and subsequent recalibrations of the volume and gas sensors were performed.

Principal ventilatory parameters such as respiratory rate (RR), expiratory tidal volume (VTex), minute ventilation (V’E), volume of oxygen consumption (V’O_2_), Volume of carbon dioxide production (V’CO_2_), fraction of the inspiratory oxygen (FIO_2_) and partial pressure of end-tidal CO_2_ (pET-CO_2_) etc. were recorded for each and every breath.

### Measurement of hemodynamic parameters

We have non-invasively measured the main hemodynamic parameters (e.g. cardiac output, stroke volume, pulse rate and mean arterial pressure etc.) via our optimised volume clamp method (ClearSight system-EV1000, Edwards Lifesciences, California, USA)^12,15,16^. As we did not observe any considerable changes in hemodynamics within first five participants, further measurements were excluded.

### Relative changes and corresponding variations

All measured variables are bound to have intra- and inter individual variations. In order to realize the relative changes in measured variables, each volunteer was used as his or her (own) control. Therefore, we normalized VOC concentrations and respiratory parameters from both setups onto the corresponding values in the second breath (from first minute) of the study setup-1.

### Statistical analysis

Sample size was determined by statistical power calculation using pre-defined analysis of variance (ANOVA) test. For a minimum detectable difference (as observed in our previous clinical studies) in mean substance intensities of 400 cps, an estimated standard deviation between 250 and 300 (i.e. varying substance wise and we used 260 as an estimated mean value) and 2 groups to attain an alpha value of 0.005 and a test power of 0.99 within a population of 100,000, the sample size turned out to be 25. Therefore, we included 25 subjects in order to detect even less than 5% differences in exhaled VOCs at low ppbV and until high pptV levels.

For statistical comparisons between different breathing patterns were done via two principle approaches:

First of all, normalised mean values (from all participants) and corresponding intra individual variations of VOC concentrations and respiratory parameters of each group were calculated (i.e. over the 180 s of measurement of each respiratory rhythm). In case of non-parametric distribution of data, median values were considered for statistical analysis in both setups. As the normalised mean/median values are influenced by each volunteer, we considered analysing the effects from individual contributions (intra-individual variations) statistically. Thus, relative standard deviations (RSDs: coefficient of variation from each subject) were calculated (i.e. over the entire 180 s of measurement of each rhythm as well as over final 60 s of steady state within each rhythm) for VOC concentrations and respiratory parameters. The RSDs (in %) were calculated by rating sample standard deviations (SDs) over corresponding individual sample mean (average of raw data from each subject over each respiratory rhythm).

Statistically significant differences in all above-mentioned parameters were judged via repeated measurement ANOVA on ranks (Friedman repeated measures analysis of variance on ranks, Shapiro–Wilk test for normal distribution and post hoc Student–Newman–Keuls method for pairwise multiple comparisons between all groups; *p*-value ≤ 0.005) in SigmaPlot (version 14) software. All spontaneous rhythms (from both setups) were compared to each other and paced rhythms were compared similarly. For spontaneous breathing, we selected those (from all pairwise comparisons) referring to the corresponding values from the final 180 s (7th–9th min) for spontaneous breathing, from the setup-1. Similarly, for paced breathing we selected those referring to the corresponding values from the middle 180 s (4th–6th min) of respiration, from the setup-1.

In order to understand the persistence or extent of unsupervised physiological effects during all spontaneous rhythms, exhaled VOC concentrations from spontaneous rhythms were compared further. These comparisons were performed over 60 s and over 30 s of intervals, respectively. V’E and pET-CO_2_ values were compared in the same way. Statistical significances were tested by means of above-mentioned ANOVA test (*p*-value ≤ 0.005).

Correlations between VOC concentrations and respiratory parameters (*p*-value ≤ 0.005) were tested via *Pearson Product Moment Correlation* analysis.

In order to model the breath-resolved ventilatory variations/fluctuations in spirometric and capnometric parameters during different respiratory rhythms, multiple linear regressions were applied onto the endogenous VOC exhalation, minute ventilation and pET-CO_2_ from setup-1. After that the measured data from setup-2 were applied onto these regression equations (viz. derived from the setup-1) to predict breath-resolved variations in V’E and pET-CO_2_ during varying respiratory rhythms in setup-2. We assessed the agreement between the independently predicted ventilatory variations in setup-2 (Fig. 6) and the actual measures via Kolmogorov–Smirnov test (one-way RM ANOVA; *p*-value ≤ 0.005).

## Supplementary information

Supplementary information.

## Data Availability

Authors comply with the data availability policy of Scientific Reports.
