# Supplementary material for: Exhaled breath compositions under varying respiratory rhythms reflects ventilatory variations: translating breathomics towards respiratory medicine
Source: Sci Rep. 2020 Aug 24;10:14109. doi: 10.1038/s41598-020-70993-0 (PMC7445240; doi:10.1038/s41598-020-70993-0)
Supplement: Supplementary file 1 — Supplementary information. [file 41598_2020_70993_MOESM1_ESM.pdf]

Exhaled breath compositions under varying respiratory rhythms reflects ventilatory variations: translating breathomics towards respiratory medicine

Pritam Sukul[1]*, Jochen K Schubert[1], Karim Zanaty[1], Phillip Trefz[1], Anupam Sinha[2], Svend Kamysek[1], Wolfram Miekisch[1]

[1]*Rostock Medical Breath Research Analytics and Technologies (ROMBAT), Dept. of Anaesthesiology and Intensive Care, University Medicine Rostock, Schillingallee 35, 18057 Rostock, Germany*

[2]*Institute for Clinical Chemistry and Laboratory Medicine, University Clinic Carl Gustav Carus, Fetscherstr. 74, 01307 Dresden, Germany*

* Corresponding author: pritam.sukul@uni-rostock.de

Supplementary Table S1: Summary output for equation (1) and (2). Corresponding principal statistical parameters and values e.g. R Square, statistical significance, *p*-values etc. are presented with each equation.

*V'E_iivs. = 0.0354 + (0.887 * V'E) - (0.789 * Isoprene) + (1.801 * Isoprene_iivs.)* **(1)**

Regression Statistics	
Multiple R	0.956
R Square	0.915
Adjusted R Square	0.912
Standard Error	0.065
Observations	108

ANOVA

	df	*SS*	*MS*	*F*	*Significance F*
Regression	3	4.755	1.585	374.427	1.429E-55
Residual	104	0.440	0.004		
Total	107	5.195			

	Coefficients	*Standard Error*	*t Stat*	*P-value*	*Lower 95%*	*Upper 95%*
Intercept	0.035	0.099	0.354	0.723	-0.162	0.233
V'E	0.887	0.053	16.550	6.630E-31	0.781	0.993
Isoprene_iivs.	1.801	0.223	8.068	1.304E-12	1.358	2.243
Isoprene	-0.789	0.093	-8.404	2.390E-13	-0.975	-0.603

$pET\text{-}CO_2\_iivs. = 0.512 + (0.0750 * V'E) - (0.469 * pET\text{-}CO_2) - (0.0788 * Isoprene) + (0.150 * Isoprene\_iivs.)$ **(2)**

Regression Statistics	
Multiple R	0.920
R Square	0.847
Adjusted R Square	0.841
Standard Error	0.013
Observations	108

ANOVA

	df	*SS*	*MS*	*F*	*Significance F*
Regression	4	0.105	0.026	142.771	4.284E-41
Residual	103	0.019	0.0001		
Total	107	0.124			

	Coefficients	*Standard Error*	*t Stat*	*P-value*	*Lower 95%*	*Upper 95%*
Intercept	0.511	0.048	10.641	2.774E-18	0.416	0.607
$pET\text{-}CO_2$	-0.469	0.060	-7.795	5.396E-12	-0.588	-0.349
V'E	0.075	0.011	6.616	1.683E-09	0.052	0.097
Isoprene	0.149	0.047	3.151	0.002	0.055	0.243
Isoprene_iivs.	-0.078	0.028	-2.806	0.005	-0.134	-0.023

***N.B*:** Although the coefficient of the intercept in equation (1) was statistically insignificant (*p*-value = 0.723), we could not exclude it from our model. In principle, an insignificant intercept denotes to the fact that if other predictor variables are zero (0), the constant is not significantly different from zero. As we performed these experiments on consciously living and healthy humans, we can’t assume any of our predictor variables to be zero. Thus, we preferred not to force the residuals to have a zero mean.

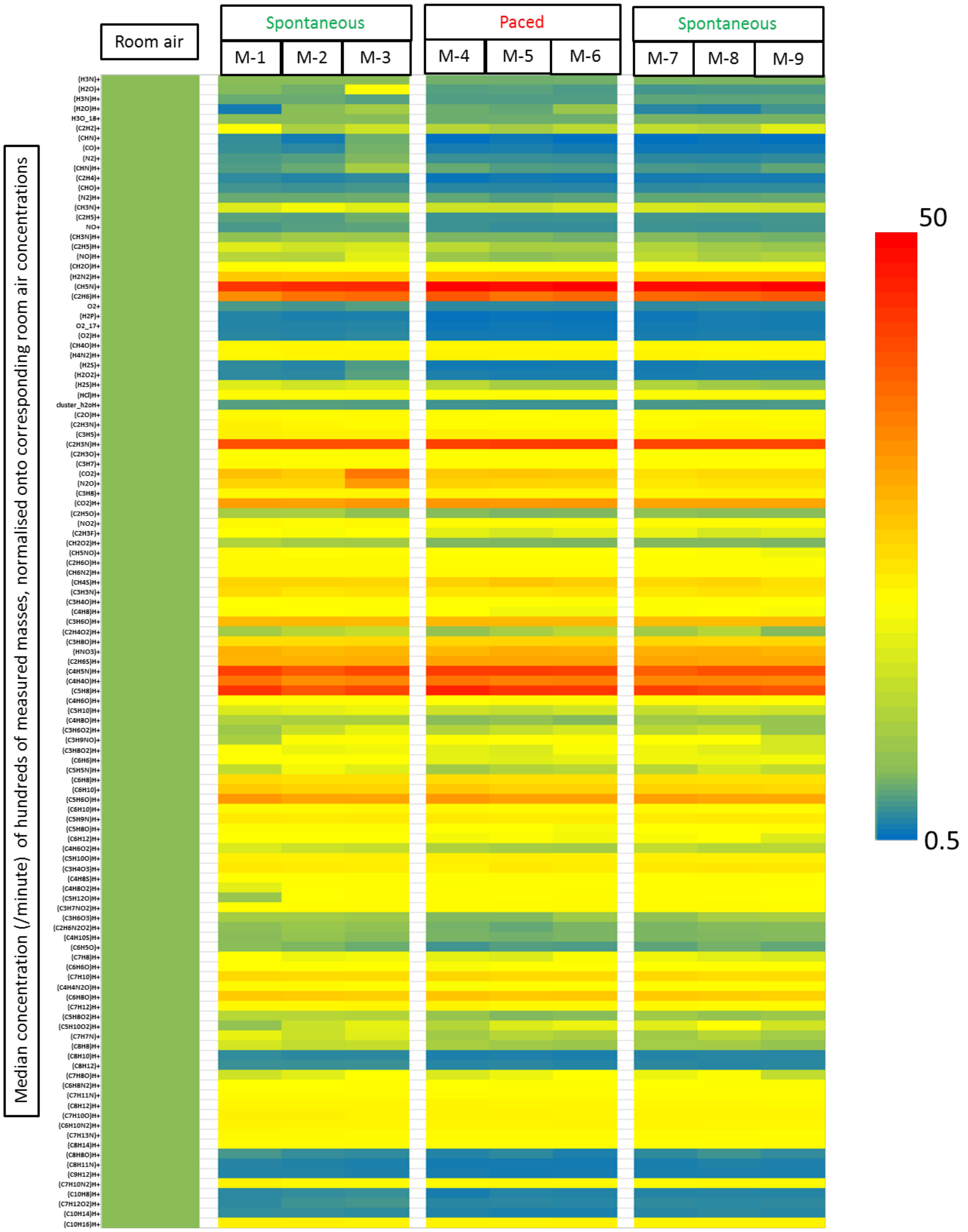

Supplementary Figure S1: Heatmap of median concentrations of hundreds of measured masses from Setup-1. Median values are calculated over each minute (from M-1 to M-9). In order to show the difference between expiratory and inspiratory abundances, exhaled concentrations are normalised onto corresponding room air (i.e. inspiratory) concentrations. This clearly shows the compounds that are contributed from the experimental environment. For each mass, the light green colour represents the room air concentration (i.e. normalisation point). With respect to the light green (i.e. room air concentrations), the yellow and red color symbolise relatively high and higher concentrations, respectively. Similarly, the dark green and blue colour represent relatively low and lower concentrations, respectively. Please refer to colour scale.

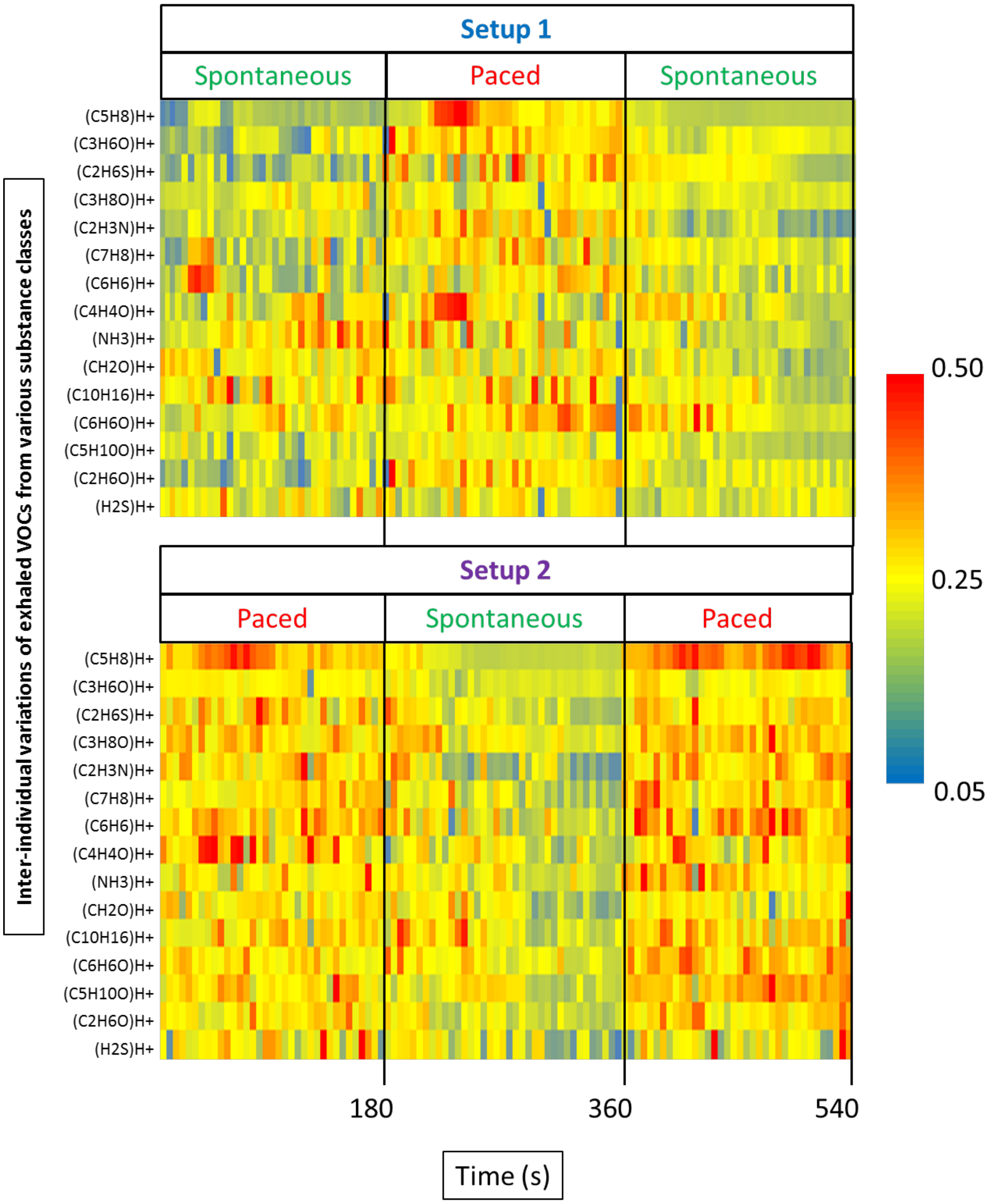

Supplementary Figure S2: Breath-resolved ventilatory variations in exhaled VOC concentrations from Setup-1 and setup-2. Changes in colours from red to blue symbolise relative changes from higher to lower values and vice versa. Please refer to the colour scale. A precedent paced breathing has substantially reduced inter-individual variations in exhaled alveolar VOC concentrations during the final minutes of subsequent spontaneous rhythms in both setups. These values are inter-individual variations (breath-resolved) of the normalised mean (from all participants) of each variable. These VOCs belong to various substance classes and origin. Substances with similar physico-chemical properties behaved alike. Statistical validation and inter-setup reproducibility of ventilatory variations are presented in Supplementary Figure S3 online.

Study setup	Respiratory rhythms	Minute	Changes (%) in median range of variations in 67 masses/60 s	Significance ($P \leq 0.005$)/ Median/60 s
**Setup-1**	Spontaneous	M1	~ 6 - 21 (+)	<0.001
		M2	~ 4.3 - 11 (±)	<0.005
		M3	~ 6 - 15 (±)	<0.001
	Paced	M4	~ 4 - 18 (+)	<0.001
		M5	~ 11 - 23 (+)	<0.001
		M6	~ 9 - 31 (+)	<0.001
	Spontaneous	M7	~ 4 - 11 (+)	<0.005
		M8	~ 2.4 - 7.3 (±)	0.005
		**M9**	**0**	**N/A**
**Setup-2**	Paced	M1	~ 5 - 25 (+)	<0.001
		M2	~ 11 - 37 (+)	<0.001
		M3	~ 17 - 45 (+)	<0.001
	Spontaneous	M4	~ 3.4 - 11 (+)	<0.005
		M5	~ 3 - 7 (±)	0.005
		**M6**	**0.5 - 2.4 (±)**	**>0.005**
	Paced	M7	~ 5 - 35 (+)	<0.001
		M8	~ 9 - 46 (+)	<0.001
		M9	~ 13 - 59 (+)	<0.001

Supplementary Table S2: Reproducibility and statistical validation of breath-resolved ventilatory variations in exhaled VOC concentrations in Setup-1 and setup-2. Differences in ventilatory variations in volatile masses were statistically compared for 67 measured volatile masses with relatively higher abundances in exhalation than in the room-air (chosen from **Supplementary Figure S1 online**). Intra-individual variations from respiratory rhythms (over each 60 s) were compared within- and between two setups. Changes (%) in median range (±) of variations in all volatiles are presented with respect to the values at the 9th minute of spontaneous rhythm from setup-1 (M9). Statistical significance of differences was tested by means of one-way repeated measurement-ANOVA on ranks (*p*-value $\leq$ 0.005). We used the 9th minute (M9 marked in bold) of the Setup-1 as the comparison point. From all pairwise-multiple comparisons, statistically different and indifferent (i.e. with no significant differences and thereby, reproducible) respiratory phases are presented with corresponding *p*-value. RM-ANOVA results of all pairwise multiple comparisons are presented in Supplementary Table S3 online.

Analysis: In our large data matrix consists of 67 rows and 18 columns. Each row represents the median values (over each minute) of variations from a mass over the entire experiment. Each column represents a particular minute of measurement. In order to statistically compare the minutes upon the variations contributed by 67 measured masses, we applied Friedman Repeated Measures Analysis of Variance on Ranks (RM-ANOVA Pairwise Multiple Comparisons via Student-Newman-Keuls Method, p-value ≤ 0.005) in SigmaPlot (version 14) software.

Friedman Repeated Measures Analysis of Variance on Ranks

**Group**	**N**	**Missing**	**Median**	**25%**	**75%**
S1-M1	67	0	0.300	0.200	0.400
S1-M2	67	0	0.300	0.200	0.400
S2-M3	67	0	0.300	0.200	0.400
S1-M4	67	0	0.300	0.200	0.500
S1-M5	67	0	0.300	0.200	0.500
S1-M6	67	0	0.300	0.200	0.500
S1-M7	67	0	0.300	0.200	0.500
S1-M8	67	0	0.300	0.200	0.400
S1-M9	67	0	0.300	0.200	0.400
S2-M1	67	0	0.300	0.200	0.500
S2-M2	67	0	0.300	0.200	0.500
S2-M3	67	0	0.300	0.200	0.500
S2-M4	67	0	0.300	0.200	0.500
S2-M5	67	0	0.300	0.200	0.500
S2-M6	67	0	0.300	0.200	0.400
S2-M7	67	0	0.300	0.200	0.500
S2-M8	67	0	0.300	0.200	0.500
S2-M9	67	0	0.300	0.200	0.500

Chi-square= 269.096 with 17 degrees of freedom. (p =<0.001)

The differences in the median values among the treatment groups are greater than would be expected by chance; there is a statistically significant difference (p =<0.001)

To isolate the group or groups that differ from the others use a multiple comparison procedure.

All Pairwise Multiple Comparison Procedures (Student-Newman-Keuls Method):

**Comparison**	**Diff of Ranks**	**q**	***p***	**Significance (*p* =<0.005)**
S2-M7 vs S1-M8	360.000	8.238	0.003	Yes
S2-M7 vs **S2-M6**	334.500	8.093	0.002	Yes
S2-M7 vs **S1-M9**	321.000	8.237	<0.001	Yes
S2-M7 vs S1-M2	317.000	8.660	<0.001	Yes
S2-M7 vs S1-M7	259.500	7.578	<0.001	Yes
S2-M7 vs S1-M1	252.500	7.921	<0.001	Yes
S2-M7 vs S2-M3	211.500	7.166	<0.001	Yes
S2-M7 vs S1-M4	187.500	6.907	<0.001	Yes
S2-M7 vs S2-M5	187.500	7.566	<0.001	Yes
S2-M7 vs S2-M4	179.000	7.985	<0.001	Yes
S2-M7 vs S1-M6	143.000	7.132	<0.001	Yes
S2-M7 vs S2-M8	115.500	6.532	<0.001	Yes
S2-M7 vs S1-M5	105.500	6.889	<0.001	Yes
S2-M7 vs S2-M1	97.500	7.534	<0.001	Yes

S2-M7 vs S2-M3	52.500	4.968	0.002	Yes
S2-M7 vs S2-M9	18.500	2.260	0.246	No
S2-M7 vs S2-M2	16.500	2.851	0.044	Do Not Test
S2-M2 vs S1-M8	343.500	8.310	0.002	Yes
S2-M2 vs **S2-M6**	318.000	8.160	<0.001	Yes
S2-M2 vs **S1-M9**	304.500	8.318	<0.001	Yes
S2-M2 vs S1-M2	300.500	8.776	<0.001	Yes
S2-M2 vs S1-M7	243.000	7.623	<0.001	Yes
S2-M2 vs S1-M1	236.000	7.997	<0.001	Yes
S2-M2 vs S2-M3	195.000	7.183	<0.001	Yes
S2-M2 vs S1-M4	171.000	6.900	<0.001	Yes
S2-M2 vs S2-M5	171.000	7.628	<0.001	Yes
S2-M2 vs S2-M4	162.500	8.105	<0.001	Yes
S2-M2 vs S1-M6	126.500	7.154	<0.001	Yes
S2-M2 vs S2-M8	99.000	6.465	<0.001	Yes
S2-M2 vs S1-M5	89.000	6.877	<0.001	Yes
S2-M2 vs S2-M1	81.000	7.665	<0.001	Yes
S2-M2 vs S2-M3	36.000	4.398	0.005	Yes
S2-M2 vs S2-M9	2.000	0.346	0.807	Do Not Test
S2-M9 vs S1-M8	341.500	8.763	<0.001	Yes
S2-M9 vs **S2-M6**	316.000	8.632	<0.001	Yes
S2-M9 vs **S1-M9**	302.500	8.834	<0.001	Yes
S2-M9 vs S1-M2	298.500	9.364	<0.001	Yes
S2-M9 vs S1-M7	241.000	8.166	<0.001	Yes
S2-M9 vs S1-M1	234.000	8.620	<0.001	Yes
S2-M9 vs S2-M3	193.000	7.788	<0.001	Yes
S2-M9 vs S1-M4	169.000	7.539	<0.001	Yes
S2-M9 vs S2-M5	169.000	8.429	<0.001	Yes
S2-M9 vs S2-M4	160.500	9.077	<0.001	Yes
S2-M9 vs S1-M6	124.500	8.130	<0.001	Yes
S2-M9 vs S2-M8	97.000	7.495	<0.001	Yes
S2-M9 vs S1-M5	87.000	8.233	<0.001	Yes
S2-M9 vs S2-M1	79.000	9.651	<0.001	Yes
S2-M9 vs S2-M3	34.000	5.874	<0.001	Yes
S2-M3 vs S1-M8	307.500	8.400	<0.001	Yes
S2-M3 vs **S2-M6**	282.000	8.236	<0.001	Yes
S2-M3 vs **S1-M9**	268.500	8.423	<0.001	Yes
S2-M3 vs S1-M2	264.500	8.962	<0.001	Yes
S2-M3 vs S1-M7	207.000	7.625	<0.001	Yes
S2-M3 vs S1-M1	200.000	8.070	<0.001	Yes
S2-M3 vs S2-M3	159.000	7.093	<0.001	Yes
S2-M3 vs S1-M4	135.000	6.733	<0.001	Yes
S2-M3 vs S2-M5	135.000	7.635	<0.001	Yes
S2-M3 vs S2-M4	126.500	8.261	<0.001	Yes
S2-M3 vs S1-M6	90.500	6.993	<0.001	Yes
S2-M3 vs S2-M8	63.000	5.962	<0.001	Yes
S2-M3 vs S1-M5	53.000	6.475	<0.001	Yes
S2-M3 vs S2-M1	45.000	7.775	<0.001	Yes
S2-M1 vs S1-M8	262.500	7.666	<0.001	Yes
S2-M1 vs **S2-M6**	237.000	7.435	<0.001	Yes
S2-M1 vs **S1-M9**	223.500	7.573	<0.001	Yes
S2-M1 vs S1-M2	219.500	8.085	<0.001	Yes
S2-M1 vs S1-M7	162.000	6.537	<0.001	Yes
S2-M1 vs S1-M1	155.000	6.915	<0.001	Yes
S2-M1 vs S2-M3	114.000	5.686	0.001	Yes
S2-M1 vs S1-M4	90.000	5.090	0.005	Yes

S2-M1 vs S2-M5	90.000	5.877	<0.001	Yes
S2-M1 vs S2-M4	81.500	6.297	<0.001	Yes
S2-M1 vs S1-M6	45.500	4.306	0.002	Yes
S2-M1 vs S2-M8	18.000	2.199	0.265	No
S2-M1 vs S1-M5	8.000	1.382	0.328	Do Not Test
S1-M5 vs S1-M8	254.500	7.984	<0.001	Yes
S1-M5 vs **S2-M6**	229.000	7.759	<0.001	Yes
S1-M5 vs **S1-M9**	215.500	7.938	<0.001	Yes
S1-M5 vs S1-M2	211.500	8.534	<0.001	Yes
S1-M5 vs S1-M7	154.000	6.870	<0.001	Yes
S1-M5 vs S1-M1	147.000	7.332	<0.001	Yes
S1-M5 vs S2-M3	106.000	5.995	<0.001	Yes
S1-M5 vs S1-M4	82.000	5.355	0.002	Yes
S1-M5 vs S2-M5	82.000	6.336	<0.001	Yes
S1-M5 vs S2-M4	73.500	6.955	<0.001	Yes
S1-M5 vs S1-M6	37.500	4.581	0.003	Yes
S1-M5 vs S2-M8	10.000	1.728	0.222	Do Not Test
S2-M8 vs S1-M8	244.500	8.285	<0.001	Yes
S2-M8 vs **S2-M6**	219.000	8.067	<0.001	Yes
S2-M8 vs **S1-M9**	205.500	8.292	<0.001	Yes
S2-M8 vs S1-M2	201.500	8.989	<0.001	Yes
S2-M8 vs S1-M7	144.000	7.182	<0.001	Yes
S2-M8 vs S1-M1	137.000	7.748	<0.001	Yes
S2-M8 vs S2-M3	96.000	6.269	<0.001	Yes
S2-M8 vs S1-M4	72.000	5.563	<0.001	Yes
S2-M8 vs S2-M5	72.000	6.814	<0.001	Yes
S2-M8 vs S2-M4	63.500	7.758	<0.001	Yes
S2-M8 vs S1-M6	27.500	4.751	<0.001	Yes
S1-M6 vs S1-M8	217.000	7.993	<0.001	Yes
S1-M6 vs **S2-M6**	191.500	7.727	<0.001	Yes
S1-M6 vs **S1-M9**	178.000	7.941	<0.001	Yes
S1-M6 vs S1-M2	174.000	8.678	<0.001	Yes
S1-M6 vs S1-M7	116.500	6.588	<0.001	Yes
S1-M6 vs S1-M1	109.500	7.151	<0.001	Yes
S1-M6 vs S2-M3	68.500	5.293	0.002	Yes
S1-M6 vs S1-M4	44.500	4.211	0.005	Yes
S1-M6 vs S2-M5	44.500	5.437	<0.001	Yes
S1-M6 vs S2-M4	36.000	6.220	<0.001	Yes
S2-M4 vs S1-M8	181.000	7.304	<0.001	Yes
S2-M4 vs **S2-M6**	155.500	6.937	<0.001	Yes
S2-M4 vs **S1-M9**	142.000	7.082	0.002	Yes
S2-M4 vs S1-M2	138.000	7.804	<0.001	Yes
S2-M4 vs S1-M7	80.500	5.257	0.003	Yes
S2-M4 vs S1-M1	73.500	5.679	<0.001	Yes
S2-M4 vs S2-M3	32.500	3.076	0.130	No
S2-M4 vs S1-M4	8.500	1.038	0.743	Do Not Test
S2-M4 vs S2-M5	8.500	1.469	0.299	Do Not Test
S2-M5 vs S1-M8	172.500	7.695	<0.001	Yes
S2-M5 vs **S2-M6**	147.000	7.332	<0.001	Yes
S2-M5 vs **S1-M9**	133.500	7.550	0.005	Yes
S2-M5 vs S1-M2	129.500	8.457	<0.001	Yes
S2-M5 vs S1-M7	72.000	5.563	<0.001	Yes
S2-M5 vs S1-M1	65.000	6.151	<0.001	Yes
S2-M5 vs S2-M3	24.000	2.932	0.095	Do Not Test
S2-M5 vs S1-M4	0.000	0.000	1.000	Do Not Test
S1-M4 vs S1-M8	172.500	8.604	<0.001	Yes

S1-M4 vs **S2-M6**	147.000	8.313	<0.001	Yes
S1-M4 vs **S1-M9**	133.500	8.718	<0.001	Yes
S1-M4 vs S1-M2	129.500	10.006	<0.001	Yes
S1-M4 vs S1-M7	72.000	6.814	<0.001	Yes
S1-M4 vs S1-M1	65.000	7.941	<0.001	Yes
S1-M4 vs S2-M3	24.000	4.147	0.006	Do Not Test
S2-M3 vs S1-M8	148.500	8.398	<0.001	Yes
S2-M3 vs **S2-M6**	123.000	8.032	<0.001	Yes
S2-M3 vs S1-M9	109.500	8.461	<0.001	Yes
S2-M3 vs S1-M2	105.500	9.984	<0.001	Yes
S2-M3 vs S1-M7	48.000	5.864	<0.001	Yes
S2-M3 vs S1-M1	41.000	7.084	<0.001	Yes
S1-M1 vs S1-M8	107.500	7.020	<0.001	Yes
S1-M1 vs **S2-M6**	82.000	6.336	<0.001	Yes
S1-M1 vs **S1-M9**	68.500	6.482	<0.001	Yes
S1-M1 vs S1-M2	64.500	7.880	<0.001	Yes
S1-M1 vs S1-M7	7.000	1.209	0.392	No
S1-M7 vs S1-M8	100.500	7.765	<0.001	Yes
S1-M7 vs **S2-M6**	75.000	7.097	<0.001	Yes
S1-M7 vs **S1-M9**	61.500	7.513	0.003	Yes
S1-M7 vs S1-M2	57.500	9.934	<0.001	Yes
S1-M2 vs S1-M8	43.000	4.069	0.004	Yes
S1-M2 vs **S2-M6**	17.500	8.138	<0.001	Yes
S1-M2 vs **S1-M9**	4.000	0.691	0.002	Yes
**S1-M9** vs S1-M8	39.000	4.765	0.005	Yes
**S1-M9** vs **S2-M6**	**13.500**	**2.332**	**0.099**	**Do Not Test**
S2-M6 vs S1-M8	25.500	4.406	0.002	Yes

Supplementary Table S3: Results of RM-ANOVA on ranks (for Supplementary Table S2) are presented. From all pairwise multiple comparisons, the reference phase is 9th minute of the setup-1 (**S1-M9**) and the reproducible phase is 6th minute of setup-2 (**S2-M6**). These two phases were statistically indifferent (i.e. with no significant differences and thereby, reproducible) to each other and were significantly (*p*-value ≤ 0.005) different to all other phases.

Note: The multiple comparisons on ranks do not include an adjustment for ties. A result of "Do Not Test" occurs for a comparison when no significant difference is found between the two rank sums that enclose that comparison. For example, if you had four rank sums sorted in order, and found no significant difference between rank sums 4 vs. 2, then you would not test 4 vs. 3 and 3 vs. 2, but still test 4 vs. 1 and 3 vs. 1 (4 vs. 3 and 3 vs. 2 are enclosed by 4 vs. 2: 4 3 2 1). Note that not testing the enclosed rank sums is a procedural rule, and a result of Do Not Test should be treated as if there is no significant difference between the rank sums, even though one may appear to exist.
